# Can lithium be used in the setting of clozapine commencement in patients with COVID‐19 associated neutropenia: A case report

**DOI:** 10.1002/ccr3.8758

**Published:** 2024-04-12

**Authors:** Jin Quan Eugene Tan, Jessica L. Dawson, Tarun Bastiampillai

**Affiliations:** ^1^ SA Pharmacy, Flinders Medical Centre Southern Adelaide Local Health Network Adelaide South Australia Australia; ^2^ College of Medicine and Public Health Flinders University Adelaide South Australia Australia; ^3^ Centre for Medicine Use and Safety, Faculty of Pharmacy and Pharmaceutical Sciences Monash University Parkville Victoria Australia; ^4^ Discipline of Psychiatry, College of Medicine and Public Health Flinders University Adelaide South Australia Australia; ^5^ Department of Psychiatry Monash University Clayton Victoria Australia

**Keywords:** antipsychotics, clozapine, COVID‐19, neutropenia, psychiatry

## Abstract

COVID‐19 infection may increase the likelihood of neutropenia in patients already on clozapine. In clozapine treated patients experiencing COVID‐19 associated neutropenia, adjunct therapy with lithium can be considered.

## 1 INTRODUCTION

Clozapine is the gold standard for treatment resistant schizophrenia.^1^ Patients who are prescribed clozapine, require regular monitoring for clozapine‐associated neutropenia (CAN) and clozapine‐induced agranulocytosis (CIA) sometimes referred to as severe neutropenia.^2^ A meta‐analysis estimates that the overall incidence rate of mild CAN and CIA is 3.8% and 0.9%, respectively.^3^ Mortality associated with neutropenia in clozapine‐treated patients is extremely rare (0.013%).^3^ The risk of neutropenia in clozapine‐treated patients is most pronounced in the initial year of treatment, with a reported rate of 47 per 1000 person‐years, particularly during the initiation phase.^2^, ^4^, ^5^ However, this risk diminishes significantly beyond the first year, with a roughly 8‐fold lower risk in the second year compared to the first year.^5^ The exact pathologic mechanism of CAN and CIA remains unclear. Potential mechanisms include the induction of neutrophil apoptosis by a chemically reactive nitrenium ion (clozapine metabolite) or the formation of hypochlorous acid by neutrophils VIA the NADPH/myeloperoxidase pathway.^6^ Oxidation of clozapine by hypochlorous acid results in the formation of reactive metabolites, which can bind to neutrophils causing direct toxicity or initiate an immunological reaction leading to the development of CAN or CIA.^6^

### 1.1 The hematological effects of COVID‐19

Currently, COVID‐19 infection appears to have varied effects on an individual's hematology profile, lymphopenia, lymphocytosis, leukopenia, leucocytosis, thrombocytopenia, and anemia have been reported.^7^, ^8^ A cohort study with 136 patients reported quantitative abnormalities in leukocytes (28%), lymphocyte (23%), platelets (87%), and red blood cells (30.15%) in COVID‐19 positive patients.^7^ Similar to other viral respiratory infections, lymphopenia is common in COVID‐19 and has been reported to happen alongside neutrophilia.^9^ It is suspected that lymphopenia occurs firstly due to direct infection of SARS‐Cov‐2 on T‐lymphocytes, and secondly macrophage activation, driving lymphocyte apoptosis, and necrosis through induction of inflammatory cytokines (IL‐6).^10^, ^11^ The destruction of lymphocytes in COVID‐19 patients leads to an increase susceptibility to secondary bacterial infections triggering the activation and recruitment of neutrophils.^8^ Elevated neutrophil lymphocyte ratio (NLR) and platelet lymphocyte ratio (PLR) have also been identified as predictors of poor outcomes in COVID‐19 infection.^12^, ^13^

### 1.2 Interplay between clozapine and COVID‐19

In the context of COVID‐19 there is concern that clozapine could compromise patients immune system resulting in an increase susceptibility to either contracting COVID‐19 infection or experiencing increased complications related to the infection. This concern of an increased risk of COVID‐19 infection likely stems from extrapolation of well‐known observations that patients using clozapine are more vulnerable to contracting influenza and its complications.^14^ It is uncertain whether clozapine‐treated patients face an increased risk of COVID‐19 infection. A systematic review failed to discover compelling evidence to support this claim, however, a more recent study with 732 patients found that clozapine use is associated with an 81% increased risk for COVID‐19 positivity.^15^, ^16^ While clozapine might not directly increase the risk of COVID‐19 infection, pneumonia can occur as a complication.^17^ Clozapine‐treated patients are at an increased risk of pneumonia and this is associated with high mortality.^17^ This increased risk of pneumonia in clozapine treated patients is dose‐dependent and is likely driven by side effects; notably sialorrhea, sedation, aspiration, and smoking, on the background of clozapine's immunosuppressive properties.^15^, ^17^

In relation to the hematological effects of COVID‐19, an inverse effect on neutrophils have been reported in clozapine users compared to nonusers. Overall, a systematic review found that reductions in neutrophils was consistently observed during acute COVID‐19 infection in clozapine users and none of the studies included showed the typical hematological profile commonly found in COVID‐19, suggesting that the effect was primarily clozapine related.^15^ Furthermore, it was discovered that COVID‐19 infection can result in elevated clozapine plasma levels, which could lead to increased clozapine dose‐related side effects, including neutropenia.^15^ A study has found that in long‐term clozapine‐users (≥3 years of treatment), there appears to be a relationship between clozapine and norclozapine concentration with absolute neutrophil count (ANC) independent of clozapine dose. As the ratio of clozapine/norclozapine increased, neutrophil counts decreased.^18^ Depending on the severity of lymphopenia and/or neutropenia, discontinuing clozapine may be necessary.

### 1.3 Neutrophilic properties of lithium and its implications in COVID‐19

Lithium is known to increase white cell counts (WCC) and neutrophils.^19^ Several studies have reported the use of lithium targeting a minimum serum level of 0.4 mmol/L, to manage CAN or to prevent neutropenia when rechallenging clozapine.^20^, ^21^, ^22^ The neutrophilic mechanism of lithium is not completely understood, but possible mechanisms include direct stem‐cell stimulation, an increase in granulocyte production, stimulation of cytokines, redistribution of demarginated leukocytes, and increased cortisol production.^19^, ^23^ While lithium can augment WCC to enable continuation of clozapine in the setting of CAN, it does not protect against CIA and currently there is no reliable way to identify patients who will progress from CAN to CIA.

Lithium has also been proposed as a treatment option in COVID‐19 as it exerts both antiviral and immunomodulatory effects.^24^ Preclinical studies have demonstrated that lithium can inhibit replication of various types of viruses including those belonging to coronavirus family.^25^ The antiviral mechanisms of lithium are thought to be related to inhibition of phosphatidylinositol signaling pathway, regulation of autophagy, and inhibition of glycogen kinase‐3 isoform.^24^ Results from a case report found that treatment with lithium in COVID‐19 patients is associated with a reduction in C‐reactive protein (CRP) levels along with NLR and PLR.^25^ Lithium's immunomodulating effects are thought to be related to various mechanisms including: (a) anti‐inflammatory effects secondary to inhibition of cyclooxygenase interleukin (IL)‐1β and synthesis of tumor necrosis factor‐α, IL‐2, and IL‐10, (b) pro‐inflammatory effects attributed to induction of IL‐4, IL‐6 and various pro‐inflammatory cytokines.^26^

We present a unique case report of a patient recently recommencing clozapine who had prolonged neutropenia, while having confirmed COVID‐19 infection, treated successfully with low dose lithium, enabling the continuation of clozapine.

## 2 CASE HISTORY

Mr A is an obese (BMI—30.1), nonsmoking 19‐year‐old man with history of treatment resistant schizoaffective disorder‐bipolar type. In the past, he responded positively to two previous trials of clozapine, with doses of 150 mg (over 17 days) and 75 mg (over 18 days). In both instances, clozapine had to be discontinued shortly after starting due to two different reasons: clozapine‐induced myocarditis in the first case, and asymptomatic sinus tachycardia with a mild increase in troponin T (46 ng/L) in the second case. Additionally, during his two previous trials of clozapine, neutropenia did not occur; WCC ranged from 4.36 to 11.63 × 10^9^/L (reference range: 4.0–11 × 10^9^/L), ANC ranged from 2.38 to 9.22 × 10^9^/L (reference range: 2.0–7.5 × 10^9^/L). He was admitted on this occasion due to worsening psychotic symptoms (hallucinations, mania, and grandiose delusions) despite being compliant with daily olanzapine 15 mg, chlorpromazine 50 mg, and sodium valproate 2500 mg, and no comorbid alcohol or substance abuse. Despite prior history of cardiovascular related side effects with clozapine, the decision was carefully made in conjunction with cardiology advice to retrial clozapine with close clinical monitoring. This clinical decision was based on the established history of response while on clozapine briefly and poor clinical response to other antipsychotic medications (lurasidone, zuclopenthixol, olanzapine, asenapine, amisulpride, quetiapine aripiprazole, risperidone, and paliperidone), recurrent hospitalisations, and poor quality of life.

## 3 METHODS

Upon clozapine recommencement, Mr A had no medical comorbidities and was unvaccinated against COVID‐19 by personal preference. Mr A's decision to remain unvaccinated against COVID‐19 is unrelated to any perceived medical risk COVID‐19 vaccine may specifically pose to clozapine‐treated patients.^27^ Mr A's clozapine titration was carried out in a 38‐bed acute inpatient mental health facility and during his period of admission mandatory daily COVID‐19 testing was conducted for all admitted patients irrespective of their physical health status. His baseline WCC was 3.7 × 10^9^/L, ANC was 2.3 × 10^9^/L, and no abnormalities were detected on his echocardiogram and troponin studies. Incidentally, on day 15 post‐commencing clozapine (day 15 dose – 200 mg), routine testing following standard procedures (polymerase chain reaction test) discovered that he had tested positive for COVID‐19 but remained asymptomatic throughout the infection. On day 19 (day 4 of COVID‐19 infection) his WCC was 2.95 × 10^9^/L and ANC was neutropenic at 1.2 × 10^9^/L, his CRP was normal at 3.4 mg/L (reference range: <5 mg/L). Clozapine was ceased immediately to avoid risk of agranulocytosis developing. Mr A remained mildly neutropenic for a period of 3 weeks (WCC ranged from: 2.90 to 3.32 × 10^9^/L, ANC ranged from: 1.20 to 1.80 × 10^9^/L) despite ceasing clozapine and with no ongoing evidence of COVID‐19. Other hematological markers of COVID‐19 infection such as lymphocyte and platelet counts remained relatively unchanged throughout his admission. Under the guidance of the hematologist, it was hypothesized that he likely had COVID‐19 induced neutropenia, as there was a close temporal association between the two events, with a differential diagnosis of CAN. This is supported by the fact that he had previously been on clozapine briefly (two prior occasions), without developing CAN and that the neutropenia persisted despite clozapine being discontinued for 3 weeks from the initial onset of neutropenia. In addition to ceasing clozapine on advice of the hematologist, Mr A's valproate dose was reduced from 1500 to 1000 mg and lithium at 450 mg daily was initiated for both its known neutrophilic and mood stabilizing effects (Mr A has history of recurrent manic episodes). On day 24 since clozapine cessation, lithium 450 mg was commenced and within 7 days Mr A's neutropenia resolved (WCC – 4.88 × 10^9^/L, ANC – 2.50 × 10^9^/L). Given the resolution of mild neutropenia with low‐dose lithium and elapsed time since his COVID‐19 infection, the decision was made to carefully recommence clozapine (31 days since his last clozapine dose). Mr A's total WCC and ANC remained stable with no further events of neutropenia and no abnormalities in sodium levels (139–140 mmol/L, reference range: 135–145 mmol/L) throughout his remaining admission.

## 4 CONCLUSION AND RESULTS

After 3 weeks on clozapine 225 mg (clozapine level 286 μg/L), lithium 450 mg (lithium level – 0.4 mmol/L), olanzapine 10 mg, and sodium valproate 1000 mg, Mr A had achieved significant improvement in psychotic and affective symptoms, and he was discharged from hospital. To this date (6 months) Mr A exhibited no ongoing signs of depression, mania, delusions or hallucinations, cardiac side effects (myocarditis and tachycardia), or having further episodes of neutropenia.

## 5 DISCUSSION

This case presentation confirms that COVID‐19 infection may increase the likelihood of neutropenia in patients already on clozapine.^28^, ^29^, ^30^ However, there is a notion that the immunosuppressive effects of clozapine might provide a protective effect during COVID‐19 infection, by reducing the risk of a cytokine storm occurring, which can be accompanied by neutrophilia.^31^ This case report supports the possible use of low dose lithium to manage prolonged neutropenia associated with COVID‐19 infection in patients on clozapine.^32^, ^33^, ^34^ Neutropenia associated with COVID‐19 in patients on clozapine tends to be mild and transient without evolving to agranulocytosis, with some authors suggesting the possibility of continuing clozapine if the patient has been stable on clozapine for more than 6 months, with no prior history of neutropenia and if ANC remains above 1.0 × 10^9^/L.^32^ In our case the patient had only been on clozapine for 19 days before developing neutropenia (COVID‐19 infection confirmed on day 15). Additionally, we did not observe any signs of lymphopenia and he was asymptomatic suggesting that his COVID‐19 infection was mild. CAN remained a strong alternative diagnostic differential and his clozapine levels were within normal range suggesting that neutropenia was not a result of reduced clozapine metabolism. The 4% rate of a second CAN or CIA while undergoing clozapine rechallenge with lithium co‐prescription has been reported as significantly lower compared to the comparator rate of 21% for a second CAN or CIA in patients undergoing clozapine rechallenge without lithium.^22^ Nonetheless, clinicians must remain hypervigilant when prescribing lithium for its neutrophilic properties, as there are concerns for potential masking of impending CIA which can be rarely fatal.^35^ Furthermore, introducing lithium can increase the risk of drug interactions and exposure to side effects (e.g., neurological side effects, renal dysfunction, thyroid, or parathyroid dysfunction).^20^, ^36^ Interestingly, while a small study suggests that lithium is linked to a reduction in inflammatory activity in COVID‐19, it also upregulates IL‐6, which is implicated in cytokine storm associated with COVID‐19.^26^, ^37^ This highlights the necessity for additional research on the potential risks and benefits of using lithium during COVID‐19 infection.

### 5.1 Limitations

The outcomes of this clinical scenario must be interpreted with caution, as currently there is limited knowledge regarding the use of lithium to manage COVID‐19 associated neutropenia in the setting of clozapine commencement. Moreover, in this present case report we were only able to observe the effects of lithium with clozapine in the setting of asymptomatic COVID‐19. Considering the temporal association between the onset of neutropenia and COVID‐19 infection in this scenario, ostensibly COVID‐19 was suggested to be a key factor. The true causality of neutropenia in this scenario was clouded by the presence of confounding factors such as clozapine, sodium valproate, and olanzapine; medications known to be associated with neutropenia and the inherent known increased risk of CAN in the initial period following clozapine commencement.^5^, ^23^, ^38^, ^39^ Moreover, a cohort study has suggested that concurrent use of sodium valproate is a significant additional risk factor for CAN (up to 5‐fold higher compared to clozapine alone).^39^ We were also unable to confirm if the improvements in WCC and ANC counts occurred purely because of lithium (immunomodulating and/or neutrophilic effects), temporary cessation of clozapine, or natural recovery from COVID‐19 infection.

It is important to further research the epidemiology of COVID‐19 associated neutropenia in the setting of concomitant clozapine treatment, evaluating the trajectory of neutropenia recovery and the effectiveness of various approaches including temporary clozapine cessation and/or low dose lithium coadministration. Given the worldwide high prevalence of COVID‐19 infection, this described clinical scenario may not be unusual. In this context, it is important to enable the maintenance of clozapine treatment, with careful clinical monitoring as there are no other effective medication alternatives for patients who have treatment resistant schizophrenia.

## AUTHOR CONTRIBUTIONS

**Jin Quan Eugene Tan:** Formal analysis; writing – original draft; writing – review and editing. **Jessica L. Dawson:** Writing – review and editing. **Tarun Bastiampillai:** Conceptualization; methodology; supervision; writing – original draft; writing – review and editing.

## FUNDING INFORMATION

This research did not receive any specific grant from funding agencies in the public, commercial, or not‐for‐profit sectors.

## CONSENT

Written informed consent was obtained from the patient to publish this report in accordance with the journal's patient consent policy.

## Data Availability

The data that support the findings of this study are available from the corresponding author upon reasonable request.
